# Traceability of Microplastic Fragments from Waste Plastic Express Packages Using Near-Infrared Spectroscopy Combined with Chemometrics

**DOI:** 10.3390/molecules29061308

**Published:** 2024-03-15

**Authors:** Xianshu Fu, Xiangliang Pan, Jun Chen, Mingzhou Zhang, Zihong Ye, Xiaoping Yu

**Affiliations:** 1 College of Environment, Zhejiang University of Technology, Hangzhou 310032, China; fxs@cjlu.edu.cn; 2 Zhejiang Provincial Key Laboratory of Biometrology and Inspection & Quarantine, College of Life Sciences, China Jiliang University, Hangzhou 310018, China; zmzcjlu@cjlu.edu.cn (M.Z.); zhye@cjlu.edu.cn (Z.Y.); yxp@cjlu.edu.cn (X.Y.); 3 College of Biological and Environmental Engineering, Zhejiang Shuren University, Hangzhou 310021, China; 4 Laboratory of Pollution Exposure and Health Intervention Technology, Interdisciplinary Research Academy, Zhejiang Shuren University, Hangzhou 310021, China

**Keywords:** exhaustive and parallel half-against-half (EPHAH), large-class-number classification (LCNC), microplastic (MP), near-infrared (NIR), partial least squares discriminant analysis (PLS-DA), waste plastic express packages (WPEPs)

## Abstract

The pollution from waste plastic express packages (WPEPs), especially microplastic (MP) fragments, caused by the blowout development of the express delivery industry has attracted widespread attention. On account of the variety of additives, strong complexity, and high diversity of plastic express packages (PEPs), the multi-class classification of WPEPs is a typical large-class-number classification (LCNC). The traceability and identification of microplastic fragments from WPEPs is very challenging. An effective chemometric method for large-class-number classification would be very beneficial for the comprehensive treatment of WPEP pollution through the recycling and reuse of waste plastic express packages, including microplastic fragments and plastic debris. Rather than using the traditional one-against-one (OAO) and one-against-all (OAA) dichotomies, an exhaustive and parallel half-against-half (EPHAH) decomposition, which overcomes the defects of the OAO’s classifier learning limitations and the OAA’s data proportion imbalance, is proposed for feature selection. EPHAH analysis, combined with partial least squares discriminant analysis (PLS-DA) for large-class-number classification, was performed on 750 microplastic fragments of polyethylene WPEPs from 10 major courier companies using near-infrared (NIR) spectroscopy. After the removal of abnormal samples through robust principal component analysis (RPCA), the root mean square error of cross-validation (RMSECV) value for the model was reduced to 0.01, which was 21.5% lower than that including the abnormal samples. The best models of PLS-DA were obtained using SNV combined with SG-17 smoothing and 2D (SNV+SG-17+2D); the latent variables (LVs), the error rates of Monte Carlo cross-validation (ERMCCVs), and the final classification accuracies were 6.35, 0.155, and 88.67% for OAO-PLSDA; 5.37, 0.103, and 87.33% for OAA-PLSDA; and 3.12, 0.054, and 96.00% for EPHAH-PLSDA. The results showed that the EPHAH strategy can completely learn the complex LCNC decision boundaries for 10 classes, effectively break the tie problem, and greatly improve the voting resolution, thereby demonstrating significant superiority to both the OAO and OAA strategies in terms of classification accuracy. Meanwhile, PLS-DA further maximized the covariance and data interpretation abilities between the potential variables and categories of microplastic debris, thereby establishing an ideal performance identification model with a recognition rate of 96.00%.

## 1. Introduction

The concept of microplastics (MPs), defined as plastic debris with particles <5 mm in size, was first proposed in 2004 by Thompson et al. [1] from the University of Plymouth, who published a paper on plastic debris in marine waters and sediments in the journal Science. Microplastics (MPs) are a ubiquitous form of plastic waste that have polluted contemporary environmental systems and left behind technological fossils of human activities for centuries. The prevalence of MPs in marine environments [2,3] has been reported since the early 1970s, and their presence in estuarine systems [4,5] and freshwater environments has been well documented [6,7]. However, the presence of MPs in the overall environment has been largely ignored until recently, with the number of relevant studies currently quickly increasing on account of the ubiquity of vast quantities of plastic waste and the threats they pose to both biota systems and human health [8,9,10]. With the vigorous progress of the Internet, mobile Internet, and e-commerce in China, the booming development of new modes of commerce, such as live delivery, factory e-commerce, and community retail, has led to a blowout in the express delivery industry, which has been followed by the massive consumption of plastic express packages (PEPs). According to the statistics from the State Post Bureau of the People’s Republic of China, the business volumes of express delivery service enterprises in China were 63.52, 83.36, and 100 billion pieces in 2019, 2020, and 2021, respectively, including 12.5, 16, and 19 billion PEPs. The average annual growth rates were 25.3%, 31.2%, and 19.9%, respectively [11]. The per capita express delivery volume increased from 0.01 in 2000 to about 71 pieces in 2021. In accordance with the relevant data and statistics from the China Plastics Processing Industry Association (CPPIA), about 1.8 million tons of PEPs were discarded in 2020, including plastic films, woven bags, foam boxes, pearl bags, tape, and filled plastic, among which plastic film accounted for 73.9 percent [12]. Of this, only 1–2% was recycled [13], meaning that up to 98% of this waste either ended up in refuse landfills or was discharged into the surrounding ecological environment via mismanagement through various pathways.

The main component of PEPs is polyethylene (PE), which does not degrade, even if it exists in the natural environment for hundreds of years. Waste plastic express packages (WPEPs) remain in the environment for a long time and can be transported over a long distance [14]. The fragments of WPEPs remaining in the environment further crack into smaller microplastics through physical and chemical weathering [15]. MP fragments have a smaller volume, larger specific surface area, and stronger adsorption capacity than polychlorinated biphenyl (PCB) and polycyclic aromatic hydrocarbon (PAH) pollutants [16,17,18]. MP pollution has attracted more and more attention from the country and from individuals, and it has become the fourth largest environmental pollution source, after water pollution, air pollution, and ocean and lake pollution. Plasticizer is a type of polymer material additive, widely used in plastic products, that can improve the plasticity, flexibility, and other properties of polymer plastic materials. Phthalic acid ester (PAE), one of the most important additives, is the general name of an ester formed with phthalate, a common hormone in the living environment. There are about 30 varieties of phthalic esters, among which dioctyl phthalate (DOP) is the most significant variety.

Although only about 1–2% of PEPs are currently recycled, sorting and recycling are still important solutions to the problem of pollution from WPEPs. The rapid and efficient identification of MP fragments or plastic debris in WPEPs is the most critical step in waste plastic sorting, which is the premise for the high-value-added utilization of WPEPs [19,20,21]. PEPs are characterized as having many manufacturers, various additives, a wide variety of sources, strong complexity, and high diversity, qualities which give WPEPs the typical characteristics of large-class-number classification (LCNC). The traceability and identification of microplastic fragments (or plastic debris) from WPEPs are often very challenging.

Microplastic pollution may be one of the most widespread and persistent anthropogenic changes to Earth’s surface [22]. MPs are emerging pollutants that may play roles as mediators in the environment [23,24]. MPs can be carriers of other toxic pollutants, including both organic and inorganic pollutants [25], bacteria, and deadly viruses [23], and can quickly disperse toxic substances into the environment, thus affecting organisms. Joo et al. researched the interaction between pollutants and MPs and determined the degradation pathways of MPs and the adsorbed pollutants, especially the effects of the adsorbed pollutants on the efficiency of the membrane filtration treatment of MPs [23]. Fu et al. studied the adsorption mechanisms of organic pollutants on MPs and found that hydrophobic and electrostatic interactions, as well as other non-covalent forces, were the most common mechanisms behind the adsorption of organic pollutants by MPs [25]. Astray et al. used random forests, support vector machines, and artificial neural networks to study the adsorption capacities of different MPs to organic pollutants, and the correlation coefficient of the best selected machine learning model was greater than 0.92, indicating that these types of models could be used to quickly estimate the adsorption of organic pollutants by MPs [26]. Cid-Samamed et al. believed that the addition of mono- and bivalent salt surfactants via polymer modification could assist in trapping microplastics and achieve the aggregation of microplastics in aquatic systems [24]. At present, the methods used for the analysis and identification of MPs can be divided into the three following categories: (1) physical morphology characterization analysis methods (i.e., microscopic imaging analysis), including scanning electron microscopy (SEM), scanning electron microscopy–energy dispersive spectrometry (SEM-EDS), atomic force microscopy (AFM), and fluorescence microscopic imaging (FMI); (2) chemical component spectral analysis, including near-infrared (NIR) spectrometry, Fourier transform infrared (FTIR) spectrometry, and Raman spectroscopy; and (3) thermal analysis techniques, such as differential scanning calorimetry (DSC), pyrolytic gas chromatography–mass spectrometry (Pyr-GC/MS), and thermogravimetric analysis (TGA).

SEM can provide images of plastic-like particles with extreme clarity, steep magnification, and high-resolution surface characteristics, which facilitates the differentiation of MPs and organic particles [27]. SEM-EDS can be used for further analysis, through X-ray microanalysis, to obtain elemental composition information from the already-measured samples [28]. However, both SEM and SEM-EDS are expensive and not widely available.

The chemical compositions of microplastics can be analyzed using Pyr-GC/MS, due to their characteristic pyrolytic profile, which is generated by the thermal cracking of plastic polymers [29]. Some studies used Pyr-GC/MS to simultaneously analyze polyethylene (PE), polypropylene (PP), polystyrene (PS), polyamide (PA), polymethyl methacrylate (PMMA), and polyvinyl chloride (PVC), and compare a homemade pyrolytic spectrogram library with the commercial one [30,31]. However, the Pyr-GC/MS method carries the risk of misjudgment because different polymers may produce similar pyrolytic products. Another disadvantage of Pyr-GC/MS is that the sample quantity allowed in the machine is tiny, only 0.5 mg, which is not suitable for studying heterogeneous or complex samples, such as soil, sediment, or biological samples [32]. Moreover, only one particle can be analyzed at a time, which prohibits its use for processing large quantities of samples for large-scale sampling or conventional monitoring work.

Corradini et al. explored the application of Bayesian methods in the regression calculation of MP concentration and realized the rapid assessment of MP concentration in soil using an NIR spectrometer without extraction [33] (Corradini et al., 2019). Although the accuracy could reach 10 g/kg for low-density polyethylene (LDPE), polyethylene terephthalate (PET), and PVC microplastics in soil, the repeatability of this method needs to be further improved. Barrows et al. used micro-Fourier transform infrared (mFT-IR) spectroscopy, combined with the watershed scale method, to investigate the spatial and temporal patterns of MP concentration in the Gallatin River Basin (Montana, USA) [34]. Of the tested particles, 93% were detected to be synthetic or semi-synthetic materials. Mintenig et al. found MPs with particle sizes ranging from 50 to 150 μm in raw and drinking water using an infrared (IR) spectrometer [35]. After analysis, it was concluded that these MPs may have come from plastic packaging materials used in the purification and transportation process. The above two methods are cumbersome and impose high requirements on the operators. As with IR and NIR, the non-contact measurement from Raman spectroscopy preserves the integrity of the sample, enabling its use for the identification of MPs [36,37]. However, Raman spectroscopy is sensitive to the components and chemical pigments added to MPs, which can cause interference with the determination of polymer types [38,39].

With the development of chemometrics and other theoretical technologies, NIR spectroscopy, as a nondestructive and rapid inspection method, introduces stoichiometry to the analysis of NIR spectral data, which can prevent missing primary and secondary information contained in the spectrum and improve the efficiency and accuracy of analyses. In addition, the promulgation and implementation of environmental protection laws and regulations, both at home and abroad, have played a role in promoting NIR spectroscopy as one of the mainstream technologies for the separation and reuse of waste plastics. At present, a portable NIR spectrometer is often used to assess the level of plastic pollution in soil [40], characterize the physical and chemical properties of oils [41], classify textile fiber components [42], and detect illegal drugs quickly and accurately [43]. Although the classification accuracy of portable NIR sensors is slightly lower than that of high-performance laboratory benchtop NIR spectrometers, Riba et al. still consider them effective for quality control in the field [42]. The popularization and application of portable NIR analyzers enable the possibility of rapidly detecting and separating waste MPs.

The spectra are complex and unrecognizable to the naked eye, so it is often necessary to use chemometric analysis to ensure their traceability and classification. Chemometric methods include partial least squares (PLS) [44,45], discrimination analysis (DA) [46], support vector machine (SVM), partial least squares discrimination analysis (PLS-DA) [47], and other metrological methods. PLS analysis, under the “supervised” mode, is used to select characteristic variables through which to distinguish groups and determine the relationships between samples according to the group relationships of the known samples, so as to determine the relationships between the samples to be tested [48,49]. The PLS-DA method can establish a regression model during the “dimensionality reduction” of the data and conduct a discriminant analysis (DA) of the regression results [50]. The PLS-DA technique can also filter out noise irrelevant to classification information, better obtain the differences in information between groups, preferably predict the groups of samples, and effectively enhance the data interpretation ability and effectiveness of the model [51]. The existing chemometric methods have good discriminative effects on binary classification, but there are large discriminative errors in the LCNC of WPEPs; thus, these methods are not suitable for traceability analyses of MP fragments from WPEPs.

LCNC is one of the important tasks of machine learning and can further distinguish different unknown objects by training diverse categories. LCNC problems are usually more complex than binary classification problems, since, with the increase in categories, data overlap between different categories will increase the possibility of classification errors [52]. Binary decomposition strategies are the most popular LCNC solution technologies. One-against-one (OAO) and one-against-all (OAA) are two widely used binary decomposition methods for decomposing a primeval LCNC problem into a set of two categories of classification subproblems with low complexity and easy divide-and-rule outcomes [53]. However, the OAO and OAA methods have their own characteristics when trying to match one category with another, which leads to some shortcomings in both the OAO and OAA strategies.

For a *k*-category problem, OAO decomposition uses paired classes (*i*, *j*) for modeling, establishes a binary classifier, *B_ij_*, between each pair of classes (*i*, *j*), approximates the data partition balance of each binary classifier *B_ij_*, and subsequently generates *k*(*k −* 1)/2 parallel two-category classifiers [54]. However, in the stage of *B_ij_* training, the OAO does not take the addition of other classes outside of the class (*i*, *j*) of the sample space into account; therefore, it can only effectively predict qualified votes for (*k* − 1) basic learners, but not for others [44]. These unqualified classifiers can easily vote for a real class that is not an object class, resulting in an incorrect prediction. The decomposition strategy of OAA divides each class and all other classes into two groups, and then induces *k* parallel binary classifiers [45]. The unbalanced proportions of OAA data can cause serious classification bias for a few groups of categories.

Based on the OAO and OAA methods described above, Lei and Govindaraju [46] proposed a new binary classification strategy, half-against-half (HAH), to solve the LCNC problem. In the HAH strategy, one class group is used to evaluate another class group to avoid unqualified binary classifiers, and the *k*-category is divided into two subsets, a method which is conducive to the balance of data distribution. Although the HAH method can partially solve the problems present in both the OAO and OAA strategies, HAH still encounters the difficulty of cumulative errors caused by the hierarchical structure [55]. For *k*-category problems, if the object is classified incorrectly at any of the (*k* − 1) nodes (especially the root node) during HAH decomposition, the error will be propagated to a lower level, causing the subsequent identification of internal nodes to be meaningless. Therefore, the HAH method can only obtain a comparable classification accuracy to those of the OAO and OAA models. A novel, exhaustive and parallel half-against-half (EPHAH) decomposition method that considers the advantages and disadvantages of OAO, OAA, and HAH is proposed herein. The EPHAH method adopts parallel arrangement and exhaust decomposition for *k*-category problems, enabling it not only to avoid cumulative errors, but also to combine the discriminative ability of all the decomposed binary classifiers, facilitating the learning of complex LCNC decision boundaries. The EPHAH strategy uniformly divides k-categories into two mutually matched subsets, thus matching half of the category with the other half, so as to ensure the evaluation of the two approximately balanced class groups in each binary classifier and solve the problem of data imbalance. The multi-dimensional feature space of the WPEP microplastic data is mapped to a lower-dimensional potential space via the generation of a weighted combination of the original variables to obtain more compact feature variable data. These characteristic variable data can eliminate information redundancy, prevent overfitting, and maximize the covariance between the potential variables and microplastic classes of WPEPs, thereby establishing an EPHAH-PLSDA identification model with ideal performance [56].

## 2. Results and Discussion

### 2.1. Feasibility Analysis of the Experiment

**Figure 1:**
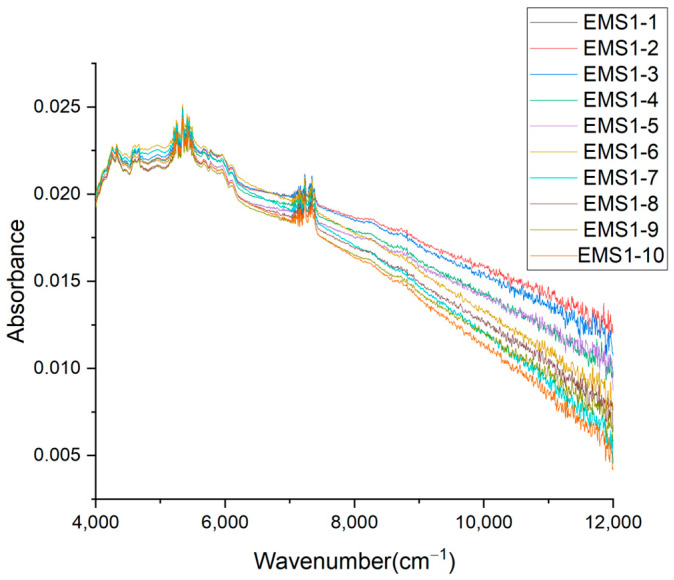
Results of 10 repeatability tests of NIR spectra of the EMS No. 1 MP sample.

In order to validate the stability and feasibility of NIR spectroscopy on microplastic particles, 10 repeatability tests were carried out on the No. 1 microplastic sample of the EMS control group under the same conditions, and the obtained NIR spectra are shown in Figure 1. Since the samples measured by the NIR spectrometer are usually un-preprocessed samples, the spectral peaks of even simple substances often overlap, as do those of complex samples. The diversity of sample information and spectral measurement signals results in the complexity, overlap, and variation of the measured spectrum, and also determines its apparent characteristics. It can be seen from Figure 1 that in 10 NIR repeatability experiments of the EMS No. 1 MP sample, the change trend of NIR spectra was consistent, indicating that the NIR spectral information obtained from 10 repeated tests was basically coherent. The characteristic peaks obtained by 10 repeated measurements were basically the same, with 12 characteristic peaks including 4250, 4323, 5245, 5338, 5390, 5440, 5700, 5773, 5940, 7010, 7230, 7340, and 7378 cm^−1^, respectively. Figure 1 demonstrates that the results of the NIR repeatability tests are satisfactory, and the peak number, peak location, and peak intensity of the spectrogram are basically the same, indicating that MP fragments from WPEPs can be detected using an NIR spectrometer.

### 2.2. NIR Spectroscopy Data Analysis of MPs from WPEPs

**Figure 2:**
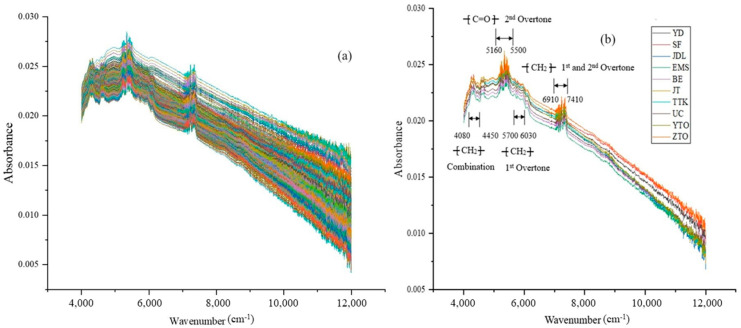
NIR spectra of 750 MP samples from 10 mainstream express delivery companies, where (**a**,**b**) are the original spectra and average spectra, respectively.

The main component of WPEP microplastics is polyethylene (PE), including both low-density and high-density polyethylene (LDPE and HDPE). In addition to polyethylene, the contents and types of auxiliary materials in the PEPs from each company are slightly different, but these differences cannot be reflected in the spectra, so there will inevitably be spectral bands with little discrepancy. The additives and main materials in the PEPs from 10 mainstream courier companies are basically the same, which is bound to bring unexpected difficulties to the correct analysis of the WPEP microplastic spectrograms. The NIR spectra of 750 MP samples from WPEPs from 10 mainstream delivery companies are shown in Figure 2, where (a) and (b) are the raw and average spectra, respectively.

It can be observed from Figure 2 that the NIR spectrum of polyethylene in MPs mainly shows the methylene (-CH2-) overtone and combination bands. The wavenumbers ranging from 4080 to 4450 cm^−1^ are the stretching and bending vibrations of the strongest peaks of the methylene band in the combination region. The wavenumber ranges from 5700 to 6030 cm^−1^ represent the asymmetric and symmetric stretching vibrations in the first overtone region of the polyethylene–methylene unit; meanwhile, the overlapping region of tensile and bending vibrations in the first and second overtone regions of the methylene band is within the wavenumber range of 6910–7410 cm^−1^. Although, theoretically, a range of 8130–8510 cm^−1^ is the symmetric stretching vibration in the second overtone region of methylene, the chromatographic peak of the spectrum is not significant. In Figure 2, the wavenumber ranges of 4080–4450, 5700–6030, and 6910–7410 cm^−1^ may be used as the methylene characteristic peaks of polyethylene in WPEP MPs to distinguish the sources of MPs and identify to which express company the PEP belongs.

Phthalic acid esters (PAEs), also known as phthalates, are esters formed from phthalic acid, and they are widely used in PE, PP, PS, and other plastics. PAEs are common environmental hormones in human life and act like estrogen in human and animal bodies. PAEs can interfere with the endocrine system, affect the male reproductive system, and increase the risk of breast cancer in women. The main functional groups are an aromatic group (-ArC-) and an ester group (R-COO-R’). In Figure 2b, the wavenumber of 5160–5500 cm^−1^ in the NIR mean spectral map is the second overtone stretching vibration region of R-COO-R’, with no significant visual difference between ester functional groups. It can be seen from Figure 2 that abundant characteristic peaks are detected in a wide range of wavenumbers, which laterally illustrates the possibility of simultaneous analyses of polyethylene and PAEs in WPEP MPs using NIR spectra.

### 2.3. Outlier Elimination

Prior to data splitting, RPCA was performed on the raw spectra (4000–12,000 cm^−1^) of the WPEP MPs to detect outliers. The abnormal sample elimination method of RPCA mainly includes the two following steps: (1) determine the number of principal components using PCA and (2) measure the mean and variance of the prediction residual. The principal component cumulative interpretation variance of 10 kinds of microplastics from waste plastic express packages is shown in Table 1. The results of the principal component analysis of the spectra can be described through cumulative interpretation variance. The higher the percentage of cumulative interpretation variance, the more spectral information is represented. It can be seen from Table 1 that with an increasing number of principal components, the cumulative interpretation variance increases correspondingly, while the range of increase becomes smaller and smaller. As shown in Table 1, for the 75 EMS MP samples, six PCs were selected to calculate the cumulative interpretation variance, which was greater than 99.9%. The inclusion of more PCs would not significantly enhance the cumulative interpretation’s variance. Similarly, the same method can be used to determine the number of PCs in the other nine classes of MPs.

**Figure 3:**
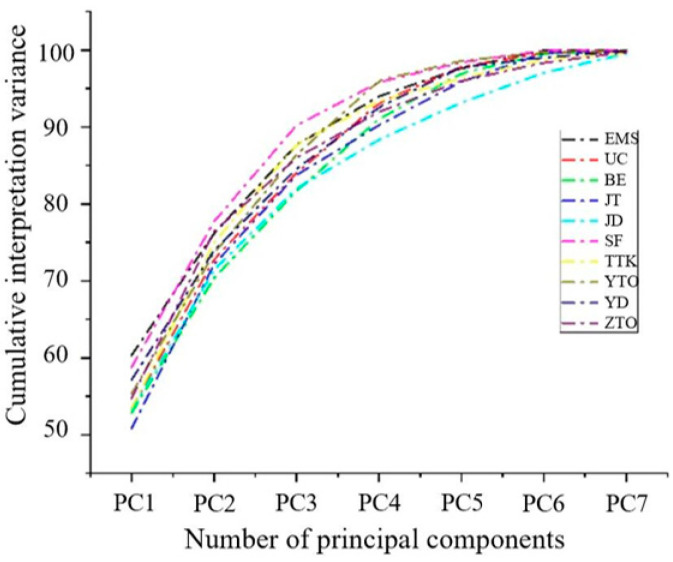
PCA interpretation diagram of 10 classes of WPEPs.

The PCA interpretation diagram of 10 classes of MP fragments is shown in Figure 3. The number of PCs can be estimated using the interpretation variance and cumulative interpretation variance. As can be seen in Table 1 and Figure 3, EMS, UC, BE, JT, SF, YD, and YTO required six PCs with a cumulative interpretation variance greater than 99.5%, while ZTO, TTK, and JD required seven PCs to achieve the same degree of interpretation variance. Based on RPCA, the OD is the measurement of spatial distance from the samples to the PCs, and the SD describes the dispersion degree of samples in the PC spatial class. Under the 95% default confidence interval, the values of SD and OD are calculated using Hotelling’s *T*^2^ test and *D*-statistic, respectively. The calculation formulas are shown in Formulas (1) and (2), as follows:

(1) $$T^{2}\left(p,n-1\right)=\frac{p\left(n-1\right)}{n-p}F\left(p,n-p\right)$$

(2) $$D_{n,p}\approx \frac{p\left(n-1\right)\left(n+1\right)}{n\left(n-p\right)}F\left(p,n-p\right)$$

where *p* and *n* are the number of principal components and samples, respectively.

Through several cycles, each sample can be entered into the prediction set, and the SD and OD values for each sample can be calculated. Then, the orthogonal outliers far away from the distribution region (low SD and high OD) and the poor orthogonal evaluation separation group values of the difference (high SD and high OD) are eliminated. The elimination of outliers from the 10 classes of microplastic fragments is shown in Table 2. EMS and UC in Table 2 reveal that outliers did not need to be removed from the EMS samples, while, for UC samples, the sample numbered UC-13 needed removal. After removal, the RMSECV value of the model was reduced to 0.01, which is 21.5% lower than that including the abnormal sample. As can be seen in Table 2, among 750 MP samples of WPEPs, 4 samples were eliminated due to outliers, among which 2 orthogonal outliers (object JT-43 and object YTO-25) and 2 outlier orthogonal evaluation separation group values (object TTK-18 and object UC-13) were detected and deleted.

### 2.4. Sample Set Division

In each data set, the SPXY data partition method was used to repeatedly divide all of the data—70% for the training set and 30% for the testing set. The number of rounds of SPXY data partition was set to 100, and the data were divided into 520 samples for the training set and 226 samples for the testing set in each round. The training and testing sets of 746 microplastic samples from WPEPs are shown in Table 3. The training set contained 52 EMS, 52 BE, 59 JT, 60 JD, 60 SF, 51 TTK, 51 UC, 48 YTO, 45 YD, and 42 ZTO objects, with the remaining objects acting as the testing set.

### 2.5. NIR Spectrogram Pretreatment

**Figure 4:**
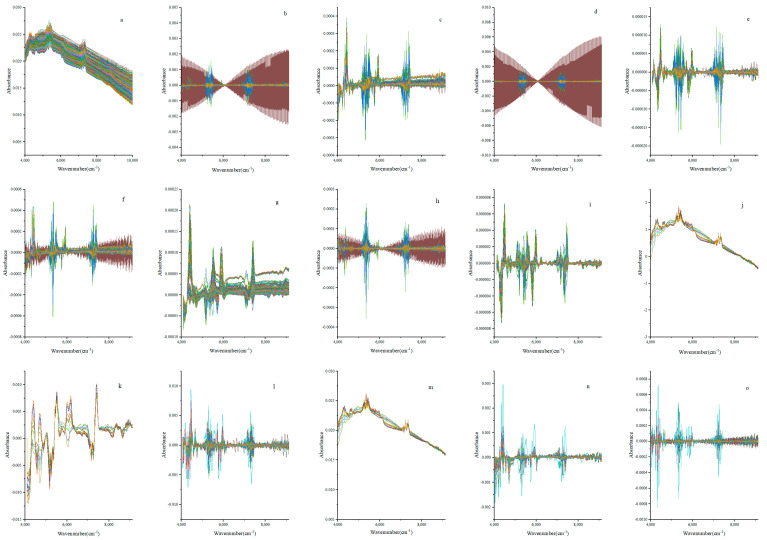
The MP samples of WPEPs were preprocessed by SG smoothing, first-order and second-order derivatives (1D and 2D), standard normal variable (SNV), and multiple scattering correction (MSC), where (**a**) SG smoothing; (**b**) 1D-smoothing one; (**c**) 1D-smoothing thirteen; (**d**) 2D-smoothing one; (**e**) 2D-smoothing twenty; (**f**) SG combined with 1D-smoothing five; (**g**) combined SG with1D-smoothing twenty-one; (**h**) combined SG to 2D-smoothing five; (**i**) associated SG with 2D-smoothing twenty-one; (**j**) SNV; (**k**) SNV combined with SG and 1D-smoothing twenty-five; (**l**) SNV combined with SG and 2D-smoothing seventeen; (**m**) MSC; (**n**) associated MSC with SG and 1D-smoothing nine; (**o**) associated MSC with SG and 2D-smoothing eight.

The NIR spectrometer can improve the signal-to-noise ratio by collecting and averaging the spectrum multiple times. Spectral noise is unavoidable, but it can be reduced or eliminated using a number of methods [50]. The single and combined pretreatment methods of Savitzky–Golay smoothing (SG), the first-order derivative (1D), the second-order derivative (2D), standard normal variable (SNV) transformation, and multiple scattering correction (MSC) are shown in Figure 4. As can be seen in Figure 4a, the noise of the spectra after SG smoothing pretreatment was somewhat reduced, but SG smoothing alone did not achieve a satisfactory effect. From Figure 4a–e, it can be seen that a low amount of SG smoothing did not eliminate the interference of noise for either the first-order or second-order derivative, while a high amount of smoothing could remove the interference from the baseline and the background. Noise interference had a satisfactory effect in the case of the first-order derivative, with 13 smoothing (1D-13 smoothing), and for the second-order derivative, with 20 smoothing (2D-20 smoothing). As seen in Figure 4f–i, SG smoothing combined with the first-order derivative (SG+1D) and SG smoothing combined with the second-order derivative (SG+2D) could effectively eliminate background interference. By comparing the derivative (D) and SG smoothing combined with the derivative (SG+D), it was obvious that SG+D had a better effect than D, and the elimination effect of SG with 21 smoothing combined with the second-order derivative (SG-21+2D) was the best. From Figure 4j–o, it can be seen that the reduction in background-induced migration from SNV transformation and MSC alone was not ideal. After SNV transformation and MSC were pretreated with SG+D, the spectral coincidence degree became higher, which weakened the impact of scattering on the original spectrum and effectively reduced the migration induced by the background. It can be seen from Figure 4 that Figure 4l had the best effect among the above 15 pretreatment methods, so the utilized pretreatment method was SNV transformation combined with SG, with 17 smoothing and the second-order derivative (SNV+SG-17+2D).

### 2.6. Characteristic Spectral Interval Selection

**Figure 5:**
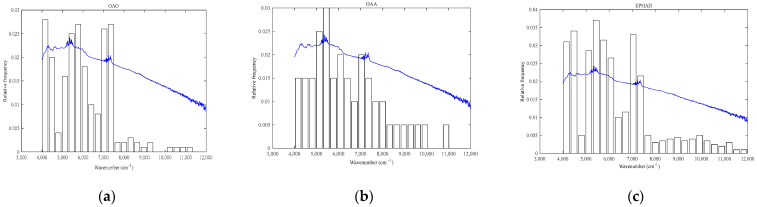
The relationship between relative frequency and wave number for each selected interval, where (**a**–**c**) represent OAO, OAA, and EPHAH, respectively, and the blue curve is the average spectrum of 746 samples.

Characteristic wavelength selection is one of the most important aspects of contemporary spectral analysis. This method can not only improve the accuracy and prediction ability of the model, but can also simplify the tracing model and accelerate the calculation and prediction speed of the model. The SI-PLS algorithm was introduced to select an NIR feature interval through which to predict the presence of PAEs and methylene in microplastic fragments. An advantage of SI-PLS is that it can represent the predictive power of each interval in a graphical display, which enables the quick and reasonable selection of spectral intervals. The SI-PLS algorithm chooses *j* intervals from *k* intervals for joint modeling, so as to select the optimal interval combination. The computational load and complexity of this method are proportional to interval number *k* and joint interval number *j*, and they increase synchronously with increases in *k* and *j*. Therefore, the value of *j* should not be too large; it is generally accepted that *j* is less than or equal to 5. In this study, the values of *j* were set at 3, 4, and 5. The wavenumber ranged from 4000 to 12,000 cm^−1^, which makes the total length of the interval 8000 cm^−1^. Considering that the wavenumber range of the overtone and combination regions of the methylene (-CH2-) and ester (-COO-) functional groups is about 300 cm^−1^, the total number of intervals of 5, 10, 15, 20, 25, and 30 were divided equally. When *k* and *j* are both equal to 5, the result is equivalent to taking the whole range of wavenumbers. There is bound to be information redundancy between each wavelength point of a continuous wavenumber range, so the model established at this time has the worst prediction effect. When *k* and *j* are equal to 25 and 5, respectively, the corresponding RMSECV value is at its minimum, reaching 0.01107, which represents the optimal interval division method. In the case of optimal interval division, the length of each interval is 320 cm^−1^. Combined with the pre-treatment method, the number of times that each interval would be selected under the three methods of OAO, OAA, and EPHAH was determined. The model parameters of OAO, OAA, and EPHAH are shown in Table 4, and the relative frequency of each selected interval is exhibited in Figure 5.

As shown in Table 4, after the spectra were pretreated using SNV, combined with SG and 17 smoothing with the second-order derivative (SNV+SG-17+2D), the parameters of the EPHAH method were better than those of the OAO and OAA models. In the EPHAH model, the average number of latent variables (LVs) and the error rate of interactive verification were 3.15 and 3.5681%, respectively, indicating that EPHAH decomposition could be used to obtain a smaller cross-verification error rate with fewer key intervals, and the model established using the EPHAH method was more accurate. The relationship between the relative frequency and spectral wavenumber in the selected intervals is shown in Figure 5. When using each method, the intervals among the top five selection times were selected as the characteristic spectral intervals. Due to the definitions of OAO, OAA, and EPHAH, 45, 10, and 126 models needed to be built, respectively, for the above three methods. In terms of Figure 5a, the top five times that were most selected using the OAO model were, respectively, 28, 27, 27, 26, and 25, and the corresponding wavenumber ranges were 4000–4320, 5600–5920, 7200–7520, 6880–7200, and 5280–5600 cm^−1^. The wavenumbers mentioned above included partial absorption peaks, in combination with the first and second overtone regions of methylene, as well as partial peaks in the second overtone region of ester. According to Figure 5b, in the case of the OAA model, the first five times that were selected were all less than 10, which could not be used as a basis for the range of characteristic spectra. For Figure 5c, using the EPHAH method, the top five most selected times were 74, 68, 66, 63, and 62, accounting for 52.8% of the total selected times, which could be determined as the required characteristic spectral intervals. The wavenumber ranges corresponding to the above characteristic spectral intervals were 5280–5600, 4320–4640, 6880–7200, 5600–5920, and 4000–4320 cm^−1^, respectively. Out of the 126 EPHAH models, the wavenumber range of 5280–5600 cm^−1^ was selected 74 times. The abovementioned wavenumbers covered the ranges of the second ester overtone region, the ester and methylene combination region, and the first and second methylene overtone regions. Methylene is the main functional group of polyethylene (PE). The combination region, the first overtone region, and the overlap region of the first and second overtones are 4080–4450, 5700–6030, and 6910–7410 cm^−1^, respectively. The ester group (R-COO-R’) is the main functional group of PAEs, for which 5160–5500 cm^−1^ is the second overtone stretching vibration region. On the basis of the comparison, in Figure 5, it can be confirmed that, among the three methods—OAO, OAA, and EPHAH—EPHAH can effectively ascertain the NIR characteristic spectral interval of microplastics. Additionally, the selected intervals are closely related to those of PE and PAEs, which are the main components of waste plastic express packages.

### 2.7. Large-Class-Number Classification Results

DUPLEX partition was adopted to process the data from each class, of which 60% were used as the training set, 20% as the verification set, and the remaining 20% as the testing set. Table 5 showed the classification of the training, verification, and testing sets of 746 microplastic samples from 10 classes of waste plastic express packages. The samples were divided into 446 training sets, 150 verification sets, and 150 testing sets for the training, verification, and testing of multi-class PLSDA models. For PLSDA models, the number of latent variables (LVs) is the key parameter, which is estimated using MCCV. The classification results of 10 classes of microplastic samples, as assessed using OAO-PLSDA, OAA-PLSDA, and EPHAH-PLSDA, are summarized in Table 6.

As can be seen in Table 6, the LVs of the OAO-PLSDA and OAA-PLSDA classifiers were 6.35 and 5.37, respectively, and the accuracy values of their testing sets were 88.67% and 87.33%. The unsatisfactory prediction capacity of the OAO-PLSDA and OAA-PLSDA models may be related to the high complexity of the models and their poor generalization performance. The final result of OAO-PLSDA was prone to overlap and sub-model errors. Although OAA-PLSDA corrected the imbalance of the data proportion, it still led to classification bias for a few groups. The results of the OAO-PLSDA and OAA-PLSDA methods were similar, while the EPHAH-PLSDA model had a higher priority than the OAO-PLSDA and OAA-PLSDA models in all indexes. As demonstrated in Table 6, for the EPHAH-PLSDA model, the total classification accuracy for the 10 classes of microplastics reached 96.00%, with average LVs of 3.12. Compared with the OAO-PLSDA and OAA-PLSDA models, the performance of the EPHAH-PLSDA model was significantly improved, which might be attributed to the lower complexity and better classification efficiency of the EPHAH-PLSDA model.

**Figure 6:**
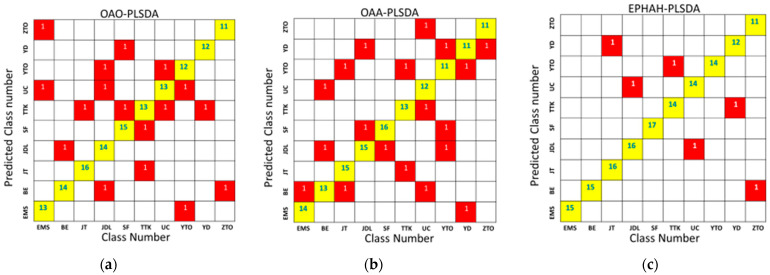
Loading diagram of PLSDA models, where (**a**–**c**) represented OAO-PLSDA, OAA-PLSDA, and EPHAH-PLSDA, respectively. In the figure, yellow and red represent correct and wrong discriminations respectively, and the numbers represent the number of discriminations.

Figure 6 shows the classification results diagram of the PLSDA models, including OAO-PLSDA, OAA-PLSDA, and EPHAH-PLSDA. According to Figure 6, in the OAO-PLSDA model, the ratio of correct classification to incorrect classification among the 150 testing sets was 133/17. One sample from each of the BE, JT, ZTO, and YD groups was misclassified as JDL, TTK, BE, and TTK. In the EMS, SF, TTK, UC, and YTO groups, two samples were misclassified in each group and, respectively, classified into UC/ZTO, TTK/YD, SF/JT, TTK/YTO, and EMS/UC. The JDL group had the most classification errors, with three samples misclassified as BE, UC, and YTO. Similarly, according to Figure 6, in the OAA-PLSDA model, 131 samples were correctly classified, and 19 samples were incorrectly classified, out of 150 testing sets. One sample from each of the EMS, SF, and ZTO groups was misclassified as BE, JDL, and YD. In the BE, JT, JDL, TTK, and YD groups, two samples in each group were misclassified into JDL/UC, BE/YTO, SF/YD, JT/YTO, and EMS/YTO. The UC and YTO groups had the most classification errors, and three samples in each group were misclassified, respectively, into BE/TTK/ZTO and JDL/SF/YD. According to Figure 6, in the EPHAH-PLSDA model, 144 samples were correctly classified and only 6 samples were wrongly classified out of 150 testing sets, demonstrating the highest accuracy among the three models. EMS, as the control group, could be perfectly separated from the other nine classes, and no other class samples were wrongly assigned to EMS. One sample from each of the JT, JDL, TTK, UC, YD, and ZTO groups was erroneously classified and assigned to YD, UC, YTO, JDL, TTK, and BE, respectively.

## 3. Materials and Methods

### 3.1. Experimental Materials

Considering that PEPs are uniformly distributed by the headquarters of each express company, we collected representative microplastic fragments or plastic debris from WPEPs from the express service points, Cainiao post stations, and garbage disposal stations in Shanghai, Hangzhou, and Nanjing, respectively. The collected fragments were clearly marked with the location and express company from which they were sourced. The sample collection information is shown in Table 7.

The number of microplastic fragments and pieces of plastic debris collected from the waste plastic express packages of 10 mainstream express companies ranged from 93 to 158 and 77 to 135, respectively. After being cleaned and dried, the collected MP fragments that were suitable for transmission were selected as the samples for detection. If the number of MP fragments meeting the NIR detection standards did not meet the requirements of this study, the collected plastic debris from WPEPs was broken down into MP fragments for testing under simulated natural weathering conditions. After the collection of 60–85 samples from each class, 750 total samples were collected, including 75 samples from China Post (EMS), 75 from Best Express (BE), 85 from Jitu Express (JT), 85 from Jingdong Express (JD), 85 from Shunfeng Express (SF), 75 from Tiantian Kuaidi Express (TTK), 75 from Yousu Express (UC), 70 from Yuantong Express (YTO), 65 from Yunda Express (YD), and 60 from Zhongtong Express (ZTO).

The 75 MP samples from EMS WPEPs were taken as the control group, and the 675 MP fragments from the remaining nine companies were used as the sample groups. The data set consisted of the EMS control group and samples from nine other courier companies (BE, JT, JD, SF, TTK, UC, YTO, YD, and ZTO). The control group and the sample groups formed the eigenmatrix X, in which the rows and columns were the referenced samples and the spectral variables, respectively, forming the eigenmatrix X of 750 × 2074. The influences of MP types and data from different companies on the modeling performance outcomes of OAA, OAO, and EPHAH were investigated through the establishment of data sets.

### 3.2. Instrument and Sample Spectral Acquisition

An NIR spectrometer (Tensor 37, Bruck, Germany) was used to collect the near-infrared diffuse reflectance spectra of MP samples with wavenumbers between 4000 and 12,000 cm^−1^. During the experiment, the samples were placed directly in a quartz cup through the collection window for spectral acquisition. With gold as the background scanning material, scans were performed 32 times at room temperature with a resolution of 8 cm^−1^ using OPUS software, version is 7.5 Build: 7, 5, 18 [20140810]. Adding to the number of scans did not significantly improve the signal-to-noise ratio or the signal. Each MP sample was detected three times to obtain the average spectrum for analysis and identification. In total, 750 NIR raw spectra were collected. The interval between data points is 3.857 cm^−1^, so each NIR spectrum has 2074 data points (variables) for a total of 2074 × 750 variables.

### 3.3. Outlier Elimination

In the process of collecting NIR spectral data, samples are affected by many factors, which can result in a large amount of irrelevant redundant information being present in the collected spectral data, and can even lead to some abnormal spectral data, called abnormal samples. Abnormal samples may be caused by factors such as the sample itself, the external shape of the sample, the instruments, the experimental operations, and other factors. The deviation between the abnormal sample data and the actual value is significantly large, and a prediction model based on these data is not credible. In order to obtain a model with better performance, it is necessary to remove abnormal samples from the spectral data. Abnormal samples are generally divided into two categories. The first category is an abnormality caused by an error in the reference value of the sample measurement; that is, the sample deviates from the corresponding space of the normal sample. The other category is a sample that is not suitable for the classification model; such samples can be identified through the evaluation parameter characteristics of a large number of models that were established statistically [51,56]. In this study, robust principal component analysis (RPCA) [52,57] was used to detect and remove outliers caused by the background, the environment, sample differences, processing, and measurement in the original spectral data set. One advantage of RPCA is that it can overcome the masking effect caused by multiple outliers. The number of principal components (PCs) was determined using cross-validation. The orthogonal distance (OD) and the scoring distance (SD) were calculated for each object using RPCA. The OD is a measure of the spatial distance from the object to the effective PC, which is related to the residual of the PCA. The SD describes the distance from the object to the center of the class, which is related to the leverage of the object. The distribution diagram of RPCA was plotted for all samples, with the OD and SD values as the axes, and samples with high values of OD or SD far away from the distribution area were eliminated from the distribution map. Each object in the RPCA distribution can be classified into the following four main categories: the normal object (with low OD and low SD), the normal scoring distance object (with low OD and high SD), the orthogonal outlier (with high OD and low SD), and the bad orthogonal evaluation separation group value (with high OD and high SD). Both the third and fourth types need to be eliminated.

### 3.4. Sample Set Division Method

The establishment of an NIR spectra analysis model using chemometrics requires sufficient and representative samples, but it is difficult to realize the practical application of such a model by manually selecting representative samples. Therefore, it is necessary to use the sample set division method to select representative samples from a large number of collected samples to establish a correction model [37]. Currently, the most commonly used sample set partitioning methods include random partitioning, the Kennard–Stone (K-S) algorithm, and the joint X–Y distance (SPXY) algorithm, based on sample set partitioning. The random partitioning method cannot ensure that the selected samples meet the requirements of the correction set. The K-S partition only considers the relationships between the spectra of the samples and ignores the relationships between the spectra and the corresponding chemical values. However, in the SPXY algorithm, Galvao et al. fully considered the relationships between spectral information and the corresponding physicochemical properties of the samples, and they calculated the joint distance between the spectra and chemical values on the basis of the K-S algorithm [58]. The SPXY algorithm can effectively cover multidimensional space and help to prevent the problem that samples with weak spectral information and low chemical values lack sensitivity to the K-S algorithm, thus validly improving the prediction performance of the model [59].

### 3.5. Spectral Pretreatment

The collected NIR spectral data mainly consist of spectral information and interference noise. This interference noise is usually generated by the instrument background, the collection environment, sample differences, and light scattering in the process of detection, issues which can easily tend to drown out spectral information [58]. In order to eliminate these causes of noise, it is often necessary to consider multiple pretreatment methods for different types of noise when choosing spectral pretreatment methods [60]. The purpose of pretreatment is to remove noise and other useless information in the spectrum, thereby improving the effect of the model. The final choice of pretreatment method is mainly determined by the effectiveness of the preprocessing method on the model.

In this study, it was indispensable to preprocess the originally collected NIR spectral data. Single pretreatment methods include Savitzky–Golay smoothing (SG smoothing) [61], standard normal variable (SNV) transformation [62], the first-order derivative (1D), the second-order derivative (2D) [63], multiple scattering correction (MSC) [47], etc. SG smoothing is a polynomial smoothing algorithm that is widely used in data denoising; proposed by Savitzky and Golag, it was based on PLS analysis and is also known as convolution smoothing. Compared with other, similar averaging methods, SG smoothing is simpler and faster, and it retains the distribution characteristics of relative maximum, minimum, and width. The first- and second-order derivatives (1D and 2D) are used to correct the baseline in order to deduce the influence of the instrument background or drift on the signal, subtract the curve background, and improve spectral resolution. SNV transformation is applied to remove spectral signal variation in order to reduce the influence of uneven particle size and non-specific scattering of the particle surface. MSC is also a commonly used data processing method in multi-wavelength calibration modeling. The basic idea is that MSC can effectively separate the absorption information of a chemical from the scattered light signal in the spectrum, assuming that the scattering coefficients are the same for all wavelengths. This method is mainly used to correct the deviation between the relative baseline translation and the NIR spectra of the samples. In this experiment, a total of 15 methods were used for spectra preprocessing, including single- and fusion-mode preprocessing algorithms.

### 3.6. Characteristic Wavelength Selection

Due to the high dimensionality and correlation characteristics of NIR, the obtained NIR spectral data are relatively vast. Moreover, the overtone and combination band absorption values of hydrogen groups at different levels in the NIR spectral region result in the serious overlap of absorption peaks, leading to a large amount of redundant, overlapping information. Therefore, the establishment of a full spectral data model not only requires a significant amount of computation, but also affects the performance of the model, due to the existence of redundant information in the spectra, which ultimately gives rise to poor prediction performance in the established model. The characteristic wavelength selection algorithm can not only reduce the dimension of spectral data and simplify the model structure, but can also effectively improve the model’s performance by selecting wavelength points with good correlation [57]. Commonly used feature band selection algorithms include the interval partial least squares (I-PLS) [64,65,66] and synergy interval partial least squares (SI-PLS) techniques [67,68,69].

The principle of the I-PLS technique is to divide the entire spectrum into smaller uniform regions, and then build a separate partial least squares regression (PLSR) [70] model for each subregion, using the same number of latent variables (LVs). First, the whole spectrum was evenly divided into *k* intervals, and then PLSR was performed on each interval to obtain *k* regression models. The root mean square error of cross-validation (RMSECV) of *k* models was calculated using the method of cross-validation, and the RMSECV value of each model was compared. The regression model corresponding to the interval with the smallest RMSECV was determined to be the optimal model. The I-PLS technique usually uses continuous wavelengths, which does not prevent the existence of redundant information between the selected wavenumbers. The SI-PLS technique is an effective feature wavelength selection algorithm, proposed by Norgaard et al. [64] and based on I-PLS. The SI-PLS technique operates sub-intervals, based on *k* intervals, divided by the I-PLS. The SI-PLS algorithm randomly selects *j* (2 ≤ *j* ≤ *k*) intervals among *k* intervals to form joint intervals with which to establish a PLS model. A total of $C_{k}^{j}$ PLS models are established, and the combination of *j* intervals corresponding to the minimum RMSECV value is the optimal interval. The calculation result of SI-PLS has a significant relationship with the values of *k* and *j*. When the value of *k* is constant, the calculation result will increase exponentially with the increase in the *j* value. Therefore, in the calculation process of SI-PLS, the value of *j* should not be too large; generally, *j* ≤ 6. The SI-PLS technique can further eliminate the redundancy of continuous wavelength intervals, a process which is conducive to the establishment of subsequent classification models and feature intervals.

### 3.7. Chemometric Analyses

The one-against-one (OAO) decomposition strategy is to divide *k*-category problems into *k*(*k* − 1)/2 parallel binary classifiers, which then pay more attention to the evaluation of one class than another [44]. For binary classifier *B_ij_* to distinguish two categories (*i*, *j*), training is conducted with the corresponding sample subset of the original data set. Category *i* is used as the training-positive object, labeled as +1, while category *j* serves as the training-negative object, marked as −1. When predicting a new object, each binary classifier allocates one of the two categories and votes on the assigned category. Combined with the decomposed parallel *k*(*k* − 1)/2 classifier, the number of votes for each category is calculated, and then the maximum voting strategy is used to predict the class of the new object, as shown in Formula (3). If there is a draw in the classification, an additional OAO model is designed with which to distinguish classes with the same maximum number of votes.

(3) $$Class=\underset{i=1,\ldots k}{\arg\max}\sum_{1\;\leq \;j\;\neq \;i\;\leq \;k}r_{OAOij}$$

where *r_OAOij_* is the predicted vote in response to *P_ij_* (*p_ji_* = −*p_ij_*) of *B_ij_*, in accordance with threshold *c* in Formula (4), and the corresponding matrix *r_OAO_* is expressed as follows:

(4) $$r_{OAOij}=\left\{\begin{array}{c} 1,\;if{\;p}_{ij}\geq c\\ 0,\;otherwise \end{array}\right.$$

The one-against-all (OAA) decomposition strategy decomposes *k*-category problems into *k* binary classification problems, each of which can be solved with a binary classifier, *B_i_*. The training of the binary classifier uses all of the training data, which are obtained through training the data of one class as the positive class, and the remaining data as the negative one. During the verification stage, it is assumed that the classifier *B_i_* is trained with the positive class of category *i*, as well as with the negative class of other categories. For a new sample X, the classifier will output a result to represent the reliability of the new sample data class. Finally, a score vector can be obtained, as follows: *P* = (*p*_1_, *p*_2_, *p*_3_, ……, *p_i_*, ……, *p_k_*). The category finally determined using the new sample X is the category with the highest credibility score, which can be expressed as follows in Formula (5):

(5) $$\mathrm{Class}=\underset{i\;=\;1,\;\ldots k}{\mathrm{argmax}}\;P_{i}$$

where *p_i_* is the predicted response value of *B_i_*.

An exhaustive and parallel half-against-half (EPHAH) decomposition method is recommended to exhaustively decompose *k*-category problems into two parallel classifiers, induced by a two-class classification partitioning strategy. The partition size of all consistent two-class classifications for *k* classes is equal to the binomial coefficient *C*(*k*, *k*/2). It should be noted that when *k* is even, only *C*(*k*, *k*/2)/2 parallel two-class classifiers need to be constructed, since the other half of the classifier is repeated. In EPHAH decomposition, each binary classifier *B_j_* is responsible for distinguishing between two divided class groups, *G_Pj_* and *G_Nj_*, which represent positive and negative objects, respectively. *k* classes are approximately split into two unified subsets (half of *k* classes against the other half), and the *k*-class problems are exhaustively resolved into *C*(*k*, *k*/2) parallel two-class classifiers. The voting *r_EPHAHij_* for the new sample is conducted as follows in Formula (6):

(6) $$r_{EPHAHij}=\left\{\begin{array}{l} 1,\;if\;p_{ij}\geq c\;and\;i\in G_{Pj}\\ 0,\;if\;p_{ij}\geq c\;and\;i\in G_{Nj}\\ 0,\;if\;p_{ij}<c\;and\;i\in G_{Pj}\\ 1,\;if\;p_{ij}<c\;and\;i\in G_{Nj} \end{array}\right.$$

As can be seen in Formula (4), if the predicted response *p_ij_* of binary classifier *B_j_* is greater than or equal to the value of threshold *c*, the trained positive objects *G_Pj_* corresponding to all classes will be voted upon. Otherwise, if *p_ij_* is lower than threshold *c*, the votes of all classes included in the trained negative objects *G_Nj_* will increase in value by 1. The maximum win voting strategy is adopted to combine the decisions of all decomposed binary classifiers, which are expressed with the matrix *r_EPHAH_*, as follows in Formula (7).

(7) $$r_{EPHAH}=\left[\begin{array}{l} r_{\mathrm{EPHAH}11}r_{EPHAH12}\cdots r_{EPHAH1C(K,K/2)}\\ r_{\mathrm{EPHAH}21}r_{EPHAH22}\cdots r_{EPHAH2C(K,K/2)}\\ {\vdots \;\;\ldots .r}_{EPHAHiC(K,K/2)}\\ r_{\mathrm{EPHAHK}1}r_{EPHAHK2}\cdots r_{EPHAHKC(K,K/2)} \end{array}\right]$$

For the new sample, its class is appointed to the one with the strongest voting power, as follows in Formula (8).

(8) $$Class=\underset{i\;=\;1,\;\ldots k}{\arg\max}\sum_{1\;\leq \;j\;\leq \;c(k,k/2)}r_{EPHAHij}$$

**Figure 7:**
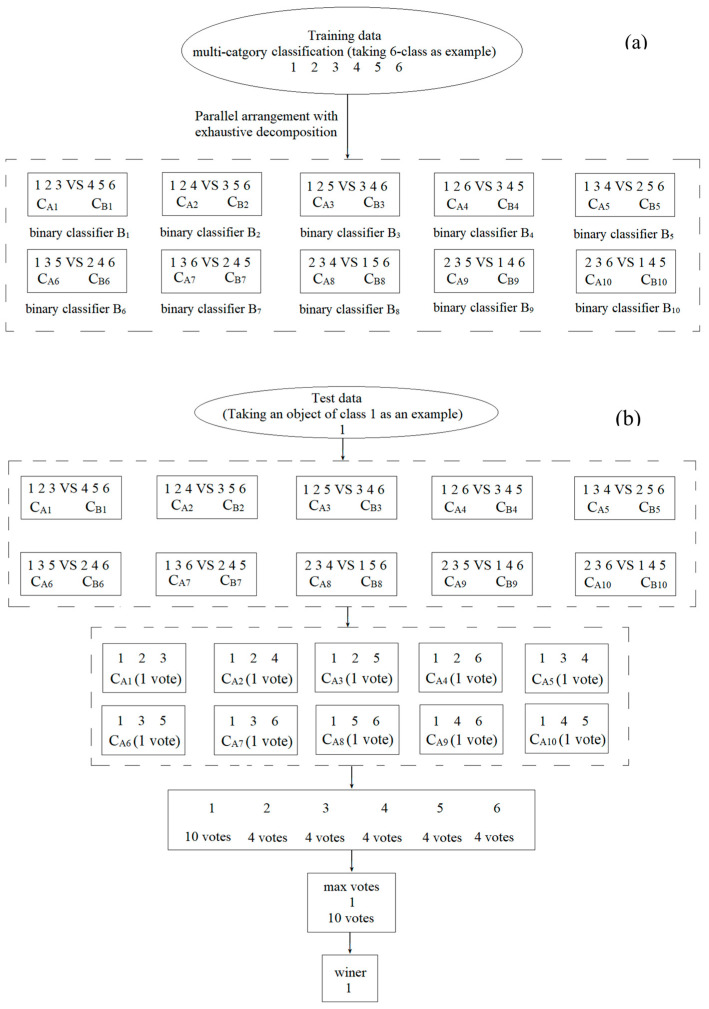
Two flow charts of the EPHAH classification problem, where (**a**,**b**) are the training phase and the test phase, respectively. The dotted box and solid line represent all parallel binary classifiers and a single specific discriminant, respectively. The arrows indicates the discriminant flow chart.

In terms of the above formula, *k* = 6 is taken as an example through which to illustrate the binarization process of EPHAH decomposition, and its flow chart is shown in Figure 7. During the training stage, as shown in Figure 7a, six classes of problems are exhaustively decomposed to generate 10 (*C*(6,3)/2) parallel binary classifiers *B_j_* (*j =* 1, 2, 3, 4, 5, 6, 7, 8, 9, 10). For example, in binary classifier *B*_1_, objects in categories 1, 2, and 3 are used to form *C_A_*_1_, and the remaining three comprise *C_B_*_1_. Two corresponding classifier models are established and distinguished through learning the differences between them. In the test phase, take category 1 in Figure 7b as an example. In binary classifier *B*_1_, if a new object’s predicted response value *p*_1_ is larger than or equal to threshold *c*, categories 1, 2, and 3 are voted upon. Otherwise, if *P*_1_ is lower than threshold *c*, categories 4, 5, and 6 are voted upon. Similarly, six categories are given votes from the other nine binary classifiers. If the classifier *B_j_* (*j =* 1, 2, 3, 4, 5, 6, 7, 8, 9, 10) makes the decision, as shown in Figure 7b, under the principle of output aggregation, the votes for the six categories are 10, 4, 4, 4, 4, and 4. According to the maximum win voting strategy, class 1, with the largest number of votes (10 votes), is marked as the prediction category of the new sample. In addition, if categories with the same number of votes are encountered during the classification process, an additional EPHAH model is established, using the categories with the same number of votes to distinguish them again.

Three different strategies, OAO, OAA, and EPHAH, were used to classify the MP sources from WPEPs of multiple categories. Both the OAO and OAA strategies use a set of binary parallel classifiers to solve the LCNC problem. For *k* groups to be analyzed, binary classifiers are established between each pair of *k* groups for the OAO strategy, resulting in a total of *k*(*k* − 1)/2 parallel OAO classifiers. All *k*(*k* − 1)/2 models use the principle of majority voting to predict and allocate the test objects. For the OAA strategy, *k* two-class (one-against-(*k* − 1)) models are constructed. For the *j* (*j* = 1, 2, 3, ……, *k*) model, the *j* group is designated as +1, and the other (*k* − 1) groups are denoted as −1. The prediction object obtains *k* predicted response vectors from the above *k* OAA models and groups them into corresponding categories, in accordance with the maximum response value. However, for the EPHAH model, the *k*-class problem is decomposed into *C*(*k*, *k*/2), a parallel two-class classifier, and the maximum winning voting strategy is adopted to assign the new sample to the class with the largest voting power.

PLS-DA is a multivariate statistical analysis method, based on the measured values of multiple variables to judge the classification of research objects. The principle is to train the characteristics of different samples, then generate the training set and, finally, test the credibility of the training set. In PLS-DA, a virtual response vector comprising +1 (*a*-class) and −1 (*b*-class) is constructed to represent each object in the eigenmatrix. The threshold of the predicted response value can be set to 0; that is, objects with a predicted response value greater than or equal to 0 are assigned to the a-class; otherwise, they are assigned to the b-class. The determination of significant latent variables (LVs) is an important key parameter of PLS-DA, as refined by Trygg et al. [71]. In this study, Monte Carlo cross-validation (MCCV) was adopted to evaluate the complexity of the model by minimizing the error rate of MCCV (ERMCCV), which is defined as follows in Formula (9):

(9) $$ERMCCV=\frac{\sum_{i=1}^{k}Mi}{N}$$

where *i* and *k* denote the lower and upper bounds, respectively. *k* is the number of random data that were split from *i* to *k* using MCCV. Mi is the total number of misjudged objects at the *i*th data split, and N is the total number of test samples among the data splits. The classification accuracy (CA) values of OAO-PLSDA, OAO-PLSDA, and EPHAH-PLSDA were evaluated and compared as shown in Formula (10):

(10) $$CA=\frac{Nc}{Nt}$$

where *N_c_* and *N_t_* are the size of the testing set and the number of correctly judged objects, respectively. The larger the ratio of *N_c_* to *N_t_*, the higher the classification accuracy (CA).

## 4. Conclusions

This study introduces the application of near-infrared characteristic fingerprinting, combined with chemometrics, to identify the sources of microplastics from waste plastic express packages. The 10 repeatability experiments using the EMS samples illustrated that the microplastic fragments from WPEPs can be detected using NIR spectroscopy, which shows good analytical ability for the simultaneous analysis of the methylene, polyethylene, and ester groups of PAEs in WPEP microplastics. The near-infrared spectral data of 750 microplastic samples were collected and analyzed using robust principal component analysis (RPCA) to eliminate abnormal samples. After removing four abnormal samples, the SPXY algorithm was introduced to divide all of the data into a training set of 520 samples and a testing set of 226 samples. Single or combination pretreatment methods, including SG smoothing, the first and second derivatives, SNV transformation, MSC, SG combined with the derivatives, SNV transformation associated with SG and the derivatives, and MSC combined with SG and the derivatives, were recommended for spectral preprocessing, and then compared according to their preconditioning effects. The pretreatment results indicated that SNV transformation combined with SG-17 smoothing and 2D (SNV+SG-17+2D) was the best preprocessing method. The synergy interval partial least squares (SI-PLS) technique was used to select the characteristic wavelength of the near-infrared spectra of microplastics. Through the relation diagram between the relative frequency and the spectral wavenumber, it was determined that the selected characteristic spectrum interval was closely related to those of polyethylene and phthalate, the main components of plastic express packages. Using the characteristic wavelength presented above, the DUPLEX partition method was adopted, according to a training set–verification set–testing set = 6:2:2, to establish the corresponding PLSDA, OAA-PLSDA, OAO-PLSDA, and EPHAH-PLSDA models. The classification accuracy values of OAO-PLSDA, OAA-PLSDA, and EPHAH-PLSDA for 10 classes of microplastic samples were 88.67%, 87.33%, and 96.00%, respectively, indicating that the EPHAH-PLSDA model greatly enhanced the performance of the large-class-number classification model, and that it had better predictive power for samples outside of the calibration data set than the OAA-PLSDA and OAO-PLSDA models. This method boosts classification performance and furnishes us with a feasible method for the geographical origin evaluation of microplastic samples from waste plastic express packages. In the future, we will combine Raman spectroscopy, X-ray diffraction, thermogravimetric analysis, and other methods to establish a rapid detection technology for microplastics using appropriate chemometric methods.

## Figures and Tables

**Table 1:** Variances in the principal component interpretation of MP samples of WPEPs.

Sample Name	Cumulative Interpretation Variance						
	PC1	PC2	PC3	PC4	PC5	PC6	PC7
EMS	60.3371	76.1853	87.6718	94.0001	97.6533	99.9848	99.9978
UC	53.3567	72.8124	84.0012	93.1071	97.8365	99.5811	99.6728
BE	52.8379	70.3580	81.6930	91.0320	96.9940	99.5114	99.9037
JT	50.8246	72.1578	83.7645	90.2234	95.8934	99.5841	99.9124
JD	53.1784	71.4354	81.9812	88.3655	93.1596	97.0560	99.5321
SF	58.7845	77.7124	90.2134	95.8125	98.3921	99.9571	99.9982
TTK	53.3485	74.9454	87.7445	93.3754	96.3187	98.7465	99.5689
YTO	55.3312	73.6155	86.3698	96.0328	98.6134	99.6752	99.9378
YD	57.1178	73.9856	84.5418	92.5794	97.7823	99.0198	99.9078
ZTO	54.7328	76.4512	85.9872	91.9633	95.9877	98.3578	99.7328

**Table 2:** Outlier elimination of 750 MP samples of WPEP.

Sample Name	Quantity of Samples	Abnormal Sample Code	PCs	Score Distance (SD)	Orthogonal Distance (OD)	Threshold Value		Type of Outlier
						SD	OD	
EMS	75	/	6	/	/	14.35	14.54	/
BE	75	/	6	/	/	14.35	14.54	/
JT	85	JT-43	6	4.20	15.42	14.16	14.32	SD small, OD large
JD	85	/	7	/		16.06	16.25	/
SF	85	/	6	/		14.16	14.33	/
TTK	75	TTK-18	7	17.45	16.96	16.38	16.60	SD large, OD large
UC	75	UC-13	6	15.54	6.78	14.35	14.54	SD large, OD small
YTO	70	YTO-25	6	2.70	15.66	14.49	14.70	SD small, OD large
YD	65	/	6	/	/	14.71	14.94	/
ZTO	60	/	7	/	/	17.07	17.35	/

**Table 3:** Training and test sets of 10 classes of MP samples of WPEPs.

Sample Name	EMS	BE	JT	JDL	SF	TTK	UC	YTO	YD	ZTO	Total Amount
Quantity	75	75	84	85	85	74	74	69	65	60	750
Training sets	52	52	59	60	60	51	51	48	45	42	520
Test sets	23	23	25	25	25	23	23	21	20	18	226

**Table 4:** Comparison of OAO, OAA, and EPHAH model parameters.

Method Name	Joint Interval Number	Average Number of Latent Variables (LVs)	Interactive Validation Error Rate
SG+2D+OAA	5	4.89	9.0034%
SG+2D+OAO	5	5.04	9.2413%
SG+2D+EPHAH	5	3.15	3.5681%

**Table 5:** Training, verification, and test set of 746 MP samples composed of 10 classes of waste plastic express packages.

Sample Name	EMS	BE	JT	JDL	SF	TTK	UC	YTO	YD	ZTO	Total Amount
Quantity	75	75	84	85	85	74	74	69	65	60	746
Training sets	45	45	50	51	51	44	44	41	39	36	446
Verification sets	15	15	17	17	17	15	15	14	13	12	150
Test sets	15	15	17	17	17	15	15	14	13	12	150

**Table 6:** The large-class-number classification results of MP samples of WPEPs by OAO-PLSDA, OAA-PLSDA, and EPHAH-PLSDA.

LCNC Model	LVs	ERMCCV	Classification Accuracy
OAO-PLSDA	6.35	0.155	0.801
OAA-PLSDA	5.37	0.103	0.792
EPHAH-PLSDA	3.12	0.054	0.950

**Table 7:** The collection information of various MP samples of WPEP.

Class Sample Name	Collection Location	Collection Region	Date	Average Size (Standard Deviation) (mm)	Average Weight (Standard Deviation) (mg)	Microplastics	Plastics	Total Microp Lastics	Total Plastics	Final Number of Samples Selected
EMS	China Post, garbage disposal stations.	(1) Shanghai: Pudong, Xuhui, and Hongkou district; (2) Hangzhou: Shangcheng, Gongshu, and Qiantang district; (3) Nanjing: Xuanwu and Baixia district.	10 May–30 December 2021	2.5(0.7)	1.3(0.5)	70	12	130	77	75
				5.5(1.8)	2.8(1.0)	60	20			
				10.3(2.1)	6.2(2.3)	0	45			
BE	BE direct stores and agency points, Cainiao courier station, and garbage disposal stations.	(1) Shanghai: Changning, Putuo, and Hongkou district; (2) Hangzhou: Xihu, Binjiang, and Qiantang district; (3) Nanjing: Qinhuai and Jianye district.	10 March–30 December 2021	3.5(1.4)	2.1(0.6)	80	0	146	93	75
				6.8(1.6)	3.4(1.5)	66	33			
				9.6(2.2)	5.8(2.7)	0	60			
JT	JT Courier station, garbage collection stations, Cainiao courier station, and courier agencies.	(1) Shanghai: Hongkou, Yangpu, Huangpu, and Jingan district; (2) Hangzhou: Shangcheng, Yuhang, Linping, and Qiantang district; (3) Nanjing: Drum Tower, Xiaguan, and Pukou district.	10 March–30 December 2021	3.7(0.6)	2.1(0.6)	67	0	149	112	85
				6.4(1.8)	3.4(1.7)	82	52			
				11.7(2.2)	6.1(2.7)	0	60			
JD	JD courier stations, garbage collection stations, Cainiao courier station, and courier agencies.	(1) Shanghai: Xuhui, Yangpu, Chongming, Jingan district; (2) Hangzhou: Qiantang, Shangcheng, Gongshu, and Xihu district; (3) Nanjing: Baixia, Qinhuai, Jianye, and Yuhuatai district.	10 March–30 December 2021	3.1(1.2)	1.9(0.8)	102	0	158	108	85
				6.2(1.5)	3.2(1.9)	56	33			
				10.5(3.1)	5.9(2.3)	0	75			
SF	SF special express stations, garbage collection stations.	(1) Shanghai: Baoshan, Minhang, and Jiading district; (2) Hangzhou: Xihu, Binjiang, Xiaoshan, and Shangcheng district; (3) Nanjing: Jianye, Gulou, and Xiaguan district.	10 March–30 December 2021	3.0(1.1)	1.8(1.3)	95	10	155	135	85
				5.5(1.2)	3.4(1.3)	60	50			
				11.5(2.5)	6.9(1.8)	0	75			
TTK	TTK special express stations, garbage collection stations, Cainiao stations, and express agents.	(1) Shanghai: Jinshan, Songjiang, and Fengxian district; (2) Hangzhou: Xihu, Gongshu, and Linping district; (3) Nanjing: Pukou, Liuhe, Qixia, Yuhuatai, and Jiangning district.	10 March–30 December 2021	2.8(1.3)	2.3(0.8)	82	0	112	114	75
				6.1(1.5)	4.8(1.3)	30	44			
				10.7(1.9)	7.3(2.0)	0	70			
UC	UC special express stations, garbage collection stations, Cainiao stations, and express agents.	(1) Shanghai: Xuhui, Hongkou, Yangpu, and Chongming district; (2) Hangzhou: Shangcheng, Gongshu, Xihu, and Binjiang district; (3) Nanjing: Baixia, Qinhuai, Jianye, and Gulou district.	10 March–30 December 2021	3.3(1.4)	2.6(0.7)	93	0	93	102	75
				6.3(1.2)	4.4(1.6)	0	52			
				11.6(2.3)	8.3(2.4)	0	50			
YTO	YTO special express stations, garbage collection stations, Cainiao stations, and express agents.	(1) Shanghai: Pudong, Huangpu, Jingan, and Songjiang district; (2) Hangzhou: Binjiang, Xihu, Xiaoshan, and Yuhang district; (3) Nanjing: Xuanwu, Gulou, Qixia, and Yuhuatai district.	10 March–30 December 2021	2.7(1.6)	3.0(1.7)	88	0	123	117	70
				6.7(2.1)	5.8(2.1)	35	47			
				11.6(2.3)	8.3(2.4)	0	70			
YD	YD special express stations, garbage collection stations, Cainiao stations, and express agents.	(1) Shanghai: Songjiang, Jiading, Jinshan, Qingpu, and Fengxian district; (2) Hangzhou: Shangcheng, Gongshu, Xihu, and Binjiang district; (3) Nanjing: Baixia, Qinhuai, Qixia, and Jianye district.	10 March–30 December 2021	4.0(1.6)	4.5(1.2)	105	0	105	102	65
				7.6(1.7)	7.8(2.5)	0	60			
				10.8(1.4)	9.2(1.7)	0	42			
ZTO	ZTO special express stations, garbage collection stations, Cainiao stations, and express agents.	(1) Shanghai: Qingpu, Fengxian, Chongming, Jingan, and Songjiang district; (2) Hangzhou: Xihu, Qiantang, Binjiang, and Xiaoshan district; (3) Nanjing: Xuanwu, Baixia, Liuhe, Qixia, and Yuhuatai district.	10 March–30 December 2021	2.9(1.3)	3.5(1.5)	82	0	125	106	60
				6.5(2.1)	7.8(2.5)	43	51			
				9.3(2.2)	8.0(1.3)	0	55			

## Data Availability

We can provide and share experimental data in order to enable other authors to achieve best practices in archiving research data.
